# Understanding older people’s intentions to use smart elderly care applications in China: an extension of the UTAUT2 model

**DOI:** 10.3389/fpubh.2025.1714110

**Published:** 2026-01-09

**Authors:** Bei Liu, Normalini Md Kassim

**Affiliations:** School of Management, Universiti Sains Malaysia, Penang, Malaysia

**Keywords:** intergenerational support, smart elderly care, technological anxiety, technology acceptance intention, UTAUT2

## Abstract

**Introduction:**

The adoption of smart elderly care applications is increasingly important in China’s aging society, yet the factors influencing older adults’ intention to use (IU) these technologies remain underexplored. This study aims to investigate these factors by extending the UTAUT2 model with additional variables, including attitude, perceived risk dimensions, intergenerational support, and technology anxiety.

**Methods:**

An online survey was conducted with 477 Chinese adults aged 60 and above, of whom 375 responses remained after quality screening. Data were analyzed using structural equation modeling (SEM) to examine the relationships between the proposed variables and IU.

**Results:**

The analysis indicates that attitude, perceived trust, and life satisfaction are significant predictors of IU. Additionally, technology anxiety exerts a notable moderating effect on these relationships.

**Discussion:**

By extending the UTAUT2 framework, this study provides deeper insights into the key factors influencing the adoption of smart elderly care applications in China. The findings offer practical implications for application designers, researchers, and government agencies seeking to promote technology adoption among older adults.

## 1 Introduction

The global population aged 65 and older is projected to double over the next three decades, reaching approximately 1.6 billion by 2050, accounting for over 16% of the total population (1). China currently has the world’s largest elderly population (2). By 2035, over 400 million individuals are projected to be aged 60 or above, exceeding 30% of the national population. This demographic shift will bring significant socio-economic challenges (3).

In response to the challenge of “aging before affluence,” the Chinese government has adopted the “9,073” care model, whereby 90% of older people are cared for by their families, 7% receive community-based services, and only 3% rely on institutional care (4). Rooted in the Confucian tradition of filial piety, family caregiving remains deeply ingrained in societal expectations, with many viewing institutional care as incompatible with filial obligations (67). Most older people also prefer aging in place, valuing the safety and comfort of familiar surroundings and social networks (5). While family-based care remains culturally preferred, demographic and social changes are placing increasing pressure on this traditional model. The long-standing one-child policy and rapid urbanization have reshaped family structures, diminishing both the availability of family caregivers and the emotional, financial, and instrumental resources accessible to older people (6).

As a result, conventional home-based care often fails to address the growing demand for professional, holistic, and personalized eldercare services. In response, smart elderly care technologies have emerged as promising alternatives (6). According to the China National Committee on Aging, smart elderly care refers to the provision of services for older people through the application of technologies such as the Internet of Things, internet-based platforms, computer information systems, and intelligent control mechanisms (65).

These innovations, enabled by advances in computational and information technologies, provide services including chronic disease management, home health monitoring, personalized health plans, online consultations, home-based assistance, and digital health records (4). Compared to traditional home or institutional care, these technologies offer scalable solutions that reduce human resource dependency and lower care-related costs.

Nevertheless, adoption of smart elderly care applications in China remains limited. Despite their functional benefits, these applications are still in their infancy, with low penetration rates. For instance, the most downloaded app has only 4.41 million downloads, representing just 1.43% of the older population (7). Understanding how and why older people develop the intention to use elderly care applications is essential, particularly given their critical role in promoting well-being during old age—an importance amplified during the COVID-19 pandemic.

Existing research on smart elderly care applications exhibits several gaps. First, most studies emphasize perceived benefits, often overlooking perceived risks, which can critically influence trust and usage intentions (8, 9). Second, while extensive research has focused on older people themselves, few studies have examined the role of intergenerational support in shaping adoption behaviors. Third, although prior studies have explored technology anxiety among older people, yet few have integrated its role with intergenerational support and perceived risks within a comprehensive adoption framework, highlighting a need for further research.

To address these gaps, this study extends the UTAUT2 model by incorporating perceived risk dimensions and integrating social exchange theory to capture intergenerational support dynamics. It further accounts for the psychological factor of technology anxiety. Together, these factors provide a comprehensive framework for analyzing the determinants and mechanisms influencing older people’s intention to use smart elderly care applications in China.

## 2 Literature review

### 2.1 Concept and research directions of smart elderly care applications

The concept of smart elderly care was initially introduced by the British Life Trust under the term “completely smart elderly care system,” emphasizing the ability of older people to enjoy high-quality lives within their own homes, free from spatial and temporal constraints (10). Smart elderly care applications refer to smartphone applications that offer functions such as chronic disease management, home health monitoring, personalized health management, online health consultations, door-to-door life care, and digital health records, all designed to support the elderly (4). Smart elderly care applications can improve the quality and efficiency of care services for older people, enhance their convenience and safety, and optimize the allocation of resources in elderly care (7).

However, existing research remains broad yet fragmented, reflecting diverse disciplinary perspectives. A large body of literature has focused on technological dimensions. For example, Shaikh et al. (11) introduced Extended Reality (XR) technologies to facilitate remote medical consultations for older people. Another major research stream focuses on smart home systems. Zhang et al. (6) traced the evolution of smart home technologies for elderly care in China, identifying key limitations and offering recommendations for future improvements. Additionally, several studies have explored sociological and ethical perspectives. Grossi et al. (12) examined the positive technology framework in elderly care, highlighting the gap between theoretical aspirations and practical implementation. Despite this growing body of research, little attention has been paid to the behavioral drivers of older people’s intention to use smart elderly care applications—an area that remains significantly underexplored.

### 2.2 Smart elderly care applications and technology acceptance theories

As emerging technologies, smart elderly care applications have been analyzed through various theoretical models, including the Technology Acceptance Model (TAM), the Unified Theory of Acceptance and Use of Technology (UTAUT), the Value-based Adoption Model (VAM), the Theory of Planned Behavior (TPB), and the extended Unified Theory of Acceptance and Use of Technology (UTAUT2). To date, most studies focusing on older people have primarily applied TAM and UTAUT, with limited use of UTAUT, despite its greater relevance for consumer contexts.

To more effectively explain consumer adoption of technology, Venkatesh Viswanath et al. (13) extended the original UTAUT model, resulting in the development of UTAUT2. This revised model preserves the foundational constructs—performance expectancy, effort expectancy, social influence, and facilitating conditions—while incorporating three new variables tailored to consumer behavior: hedonic motivation, price value, and habit. These additions broaden the model’s applicability by capturing affective and behavioral factors relevant in personal technology use.

This study adopts UTAUT2 as its theoretical framework, as it extends the original UTAUT model beyond organizational settings to capture consumer technology adoption, making it highly applicable to the context of smart elderly care in China. Importantly, UTAUT2 introduces two key factors often overlooked in prior studies on older people: hedonic motivation and price value. Hedonic motivation—defined as the enjoyment derived from using technology—has been shown to have a stronger impact on behavioral intention than performance expectancy in consumer environments. Price value, which addresses cost–benefit considerations, is particularly salient for older people evaluating technology use. Together, these two factors offer essential insights into older people’s intentions to adopt smart elderly care applications.

### 2.3 Psychological characteristics of older people

Older people often exhibit resistance to change and fear of new technologies (9). Perceived risk thus emerges as a major barrier to their adoption of smart elderly care applications. Yet, much of the existing research has focused mainly on perceived benefits, neglecting the potential risks associated with technology use. Given that technological innovations often entail both benefits and harms, integrating perceived risk into technology acceptance models is crucial for a more comprehensive understanding of adoption behavior (14). This study explicitly incorporates perceived risk, providing a rigorous and balanced analysis of older people’s intention to use smart elderly care applications.

In addition, due to age-related physiological and psychological changes, older people generally experience higher levels of technology anxiety and possess lower levels of digital literacy compared to younger individuals (15). Even if older people hold favorable attitudes toward smart elderly care and trust these technologies, technology anxiety may serve as a critical barrier to actual use. Accordingly, this study introduces technology anxiety as a moderating variable, examining its influence on attitudes, trust, and behavioral intention.

### 2.4 Intergenerational support and usage intention in the Chinese context

In China, the family continues to play a central role in caregiving for older people, serving as their most immediate and dependable source of support—particularly in Asian cultures, where strong intergenerational ties are deeply rooted in social norms (16, 17). Factors such as the number of children, household composition, and filial values have been shown to significantly influence older people’s utilization of home- and community-based services (18, 19).

Despite this, prior research has largely overlooked the influence of intergenerational support on older people’s acceptance of smart elderly care applications. Intergenerational support plays a vital role in fostering emotional closeness between parents and children. Given that life satisfaction is a core element of successful aging, it can also enhance older people’s willingness to explore and adopt new technologies (20). This study introduces intergenerational support—encompassing both emotional and financial dimensions—as a key antecedent shaping older people’s intention to adopt smart elderly care applications.

## 3 Research framework and hypotheses

**Figure 1:**
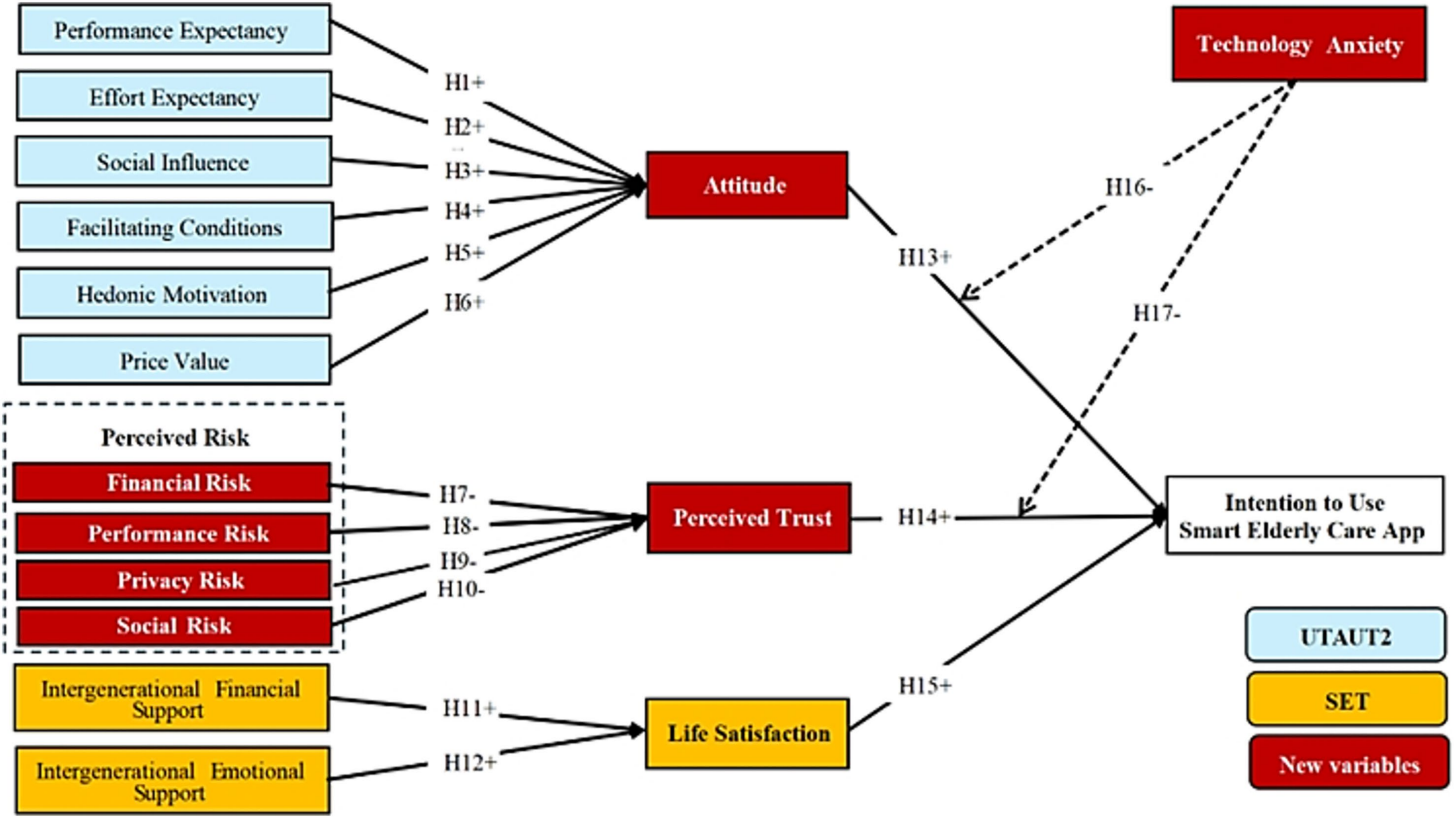
Proposed research framework.

This study extends UTAUT2 by including perceived risk, intergenerational support, attitudes, perceived trust, and life satisfaction in the framework and fully considers the moderating role of technology anxiety. Figure 1 shows the Proposed Research Framework.

Performance expectancy (PE) refers to the extent to which individuals believe that using a system will enhance their task performance (21). It is widely recognized as a key predictor of behavioral intention. When individuals perceive a technology as beneficial in daily life, they tend to form favorable attitudes toward its use (22). Numerous studies confirm the positive association between PE and attitude, particularly in the context of mobile payment technologies (22–26). In the context of smart elderly care, perceived benefits are expected to cultivate positive attitudes among older users. Thus, the following hypothesis is proposed:

*H1*: Performance expectancy (PE) is positively associated with older adults’ attitudes toward using smart elderly care applications.

Effort expectancy (EE) refers to the perceived ease of using a technology (21). Prior research has established a link between ease of use and favorable user attitudes (22, 23, 27, 28). For elderly users, especially those with limited technological literacy, perceived ease of use is crucial in shaping attitudes toward technology adoption (29). Accordingly, this study proposes:

*H2*: Effort expectancy (EE) is positively associated with older adults’ attitudes toward using smart elderly care applications.

Social influence (SI) refers to the degree to which individuals perceive that important others expect them to use a system (21). Evidence shows that influence from family, peers, and social circles positively shapes attitudes toward new technologies (13, 22, 27). Given older people’s vulnerability to the digital divide and their reliance on social networks (30), their attitudes are likely influenced by significant others (22). Thus, the following hypothesis is proposed:

*H3*: Social influence (SI) is positively associated with older adults’ attitudes toward using smart elderly care applications.

Facilitating conditions (FC) denote the availability of resources and support for system use (21). Research suggests that users with sufficient knowledge and resources are more inclined to adopt technologies (22, 23, 31). Effective implementation of smart elderly care requires coordinated support from governments, communities, service providers, and families, alongside technological infrastructure (32). Based on this, the following hypothesis is proposed:

*H4*: Facilitating conditions (FC) is positively associated with older adults’ attitudes toward using smart elderly care applications.

Hedonic motivation (HM) refers to the enjoyment or pleasure derived from technology use (13). Research shows that enjoyment significantly enhances technology acceptance (33–37). Thus, this study hypothesizes:

*H5*: Hedonic motivation (HM) are positively associated with older adults’ attitudes toward using smart elderly care applications.

Price value (PV) reflects users’ assessment of the trade-off between benefits and costs. Consumers often prioritize value in purchasing decisions (38), and studies show that high costs can dampen attitudes toward smart elderly care technologies (10, 39). Older people are more likely to adopt such technologies when they are perceived as cost-effective. Thus, the following hypothesis is proposed:

*H6*: Price value (PV) is positively associated with older adults’ attitudes toward using smart elderly care applications.

Perceived risks—including financial risk (FR), performance risk (PFR), privacy risk (PR), and social risk (SR)—can deter trust in online environments (68). Financial risk refers to potential monetary losses; performance risk involves concerns about technology performance; privacy risk relates to data security; and social risk involves potential negative judgments by others (28, 40). Prior studies suggest that increased perceived risks reduce trust in online transactions (40, 41). Accordingly, the following hypotheses are proposed:

*H7*: Financial risk (FR) is negatively associated with perceived trust.

*H8*: Performance risk (PFR) is negatively associated with perceived trust.

*H9*: Privacy risk (PR) is negatively associated with perceived trust.

*H10*: Social risk (SR) is negatively associated with perceived trust.

Intergenerational support encompasses both economic and emotional exchanges between family members (42). Financial support (IFS) involves monetary transfers, while emotional support (IES) reflects intimacy and mutual care (20). The study show that both forms of support enhance older people’s life satisfaction (42). Based on this, the following hypotheses are proposed:

*H11*: Intergenerational financial support (IFS) is positively associated with older adults’ life satisfaction.

*H12*: Intergenerational emotional support (IES) is positively associated with older adults’ life satisfaction.

Attitude (ATT), defined here as users’ evaluation of smart elderly care applications, is a core determinant of behavioral intention (27). The reasoned action approach highlights the pivotal role of attitude in shaping behavior (40). Empirical studies confirm that positive attitudes significantly predict technology adoption (23, 25, 31, 43, 44). Therefore, the following hypothesis is proposed:

*H13*: Attitude (ATT) is positively associated with the intention to use smart elderly care applications.

Perceived trust (PT) refers to individuals’ belief that a system or agent will act in their best interests (45). Trust reduces uncertainty and has been shown to enhance online transaction intentions (46). For older people unfamiliar with smart elderly care applications, trust is likely to play a critical role in adoption. Thus, the following hypothesis is proposed:

*H14*: Perceived trust (PT) is positively associated with the intention to use smart elderly care applications.

Life satisfaction (LS) reflects individuals’ overall assessment of their quality of life (42). Studies suggest that greater life satisfaction enhances openness to technology and increases the likelihood of adoption (20, 42). Thus, the following hypothesis is proposed:

*H15*: Life satisfaction (LS) is positively associated with the intention to use smart elderly care applications.

Technology anxiety (TA) describes users’ negative emotional responses, such as fear or nervousness, regarding technology use (45). Older people typically exhibit higher technology anxiety due to physical decline and lower digital literacy (27, 47). Consumers high in TA are less likely to adopt technology even if they hold favorable perceptions of it and know the benefits that can be derived from it (48, 49). This happens because they tend to focus on possible negative consequences. This factor is particularly relevant in the context of smart elderly care technology adoption.

Therefore, this study proposes the following hypothesis:

*H16*: The negative relationship between attitude (ATT) and intention to use smart elderly care applications will be stronger for more technology anxiety when compared to less technology anxiety.

*H17*: The negative relationship between perceived trust (PT) and intention to use smart elderly care applications will be stronger for more technology anxiety when compared to less technology anxiety.

## 4 Methods

### 4.1 Data source and study sample

The study shows that socioeconomics significantly affects public e-literacy, especially for older people, who are known as the “digitally disadvantaged group” (6). China’s urban development exhibits significant regional disparities, with first- and second-tier cities substantially outperforming others in terms of economic growth, infrastructure, and education levels. These cities tend to have higher levels of digital literacy among the public and more advanced supporting facilities, creating a favorable environment for the widespread adoption of smart elderly care applications (50). The retirement age of the vast majority of Chinese employees is 60, and people enter the retirement stage when they are 60 years old at the latest (51). Therefore, the samples for the study on the intention to use the smart elderly care application, which has just been developed and has a very low usage rate, are selected from the population of China’s first-tier and second-tier cities who are above 60 and have not used smart elderly care applications.

**Figure 2:**
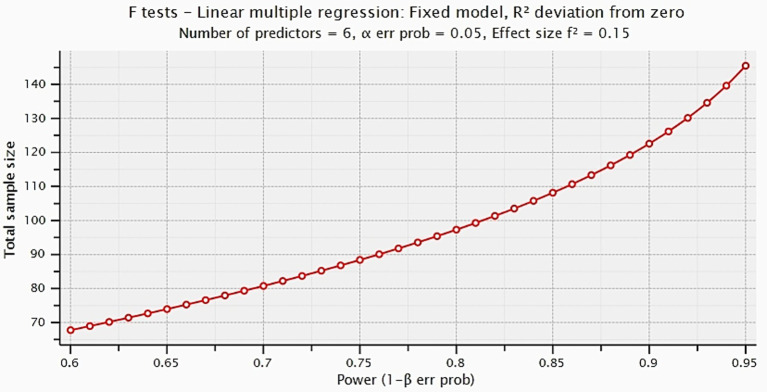
Graph of G*Power calculated data.

According to the statistical G*Power software, current research requires a minimum sample size of 146 for the present research model, assuming a significance level of 5%, an effect size of 0.15, and statistical power of 95% with 06 predictors (see Figure 2).

The more commonly used criterion for questionnaire sample size is Gorsuch’s view that it is better to keep the ratio of measurement mentions to respondents above 1:5 (52). According to this view, this questionnaire contains 69 questions, so the sample size should be greater than 345 (69*5 = 345). To collect more data, the sample size for this study was determined to be 400, which is also within the range of sample sizes suggested by many previous studies. By the end of 2023, there were 5.54 million older people aged 60 years and above in Shanghai and 2.32 million older people aged 60 years and above in Nantong. Hence, the required sample size for Shanghai city is 400*(5.54/5.54 + 2.32) = 282, and the size of the sample needed for Nantong city is 118.

With support from community workers and social media, the online survey was distributed via a link and QR code, remaining accessible until the sample size was reached. Community staff shared the survey through WeChat groups used for retirees’ “grid management”—a common governance model in China that leverages WeChat for information dissemination and community coordination. Retirees and their families typically join these groups to receive notifications regarding pension verification, medical check-ups, and government subsidies. The study indicate that approximately 79.87% of older adults in China utilize WeChat as their primary social communication tool (4). This approach enabled efficient and extensive survey distribution.

The research population consists of 477 individuals aged 60 and above living in Shanghai or Nantong city in China who have not used smart elderly care applications. The non-probability sampling method was used through the purposive sampling technique. From October 2024 to November 2024, we collected data through an online WeChat form survey of older people who have not used smart pension applications. All of the measurements were derived from the published literature.

### 4.2 Analysis and results

All questionnaire items used in this study were adapted from previously published journal articles. The two Likert scale types are applied in this study to remedy procedural errors (53). After the questionnaire design, the research started to test the validity of the content, and 3 academic experts and 3 industry experts were invited to conduct the pre-test stage to provide validity. Nine respondents participated in the pre-test stage, and the questionnaire was modified according to the opinions of experts. This study employed IBM SPSS Statistics 27.0 software for data coding, data entry, and descriptive analysis. The model measurement and assessment analysis were done through the computation of the data in SmartPLS 4.1.0.3.

#### 4.2.1 Demographic profile

The initial screening contains blank responses screening, straight lining, checking missing values. SPSS version 27 was used in this study to determine the variance of the straight line questions. Four hundred and seventy-seven cases were evaluated, of which 102 were excluded from further analysis. Three hundred seventy-five cases remained valid after being deleted by Straight Lining.

This study aimed to eliminate CMV by examining its impact on questionnaire design and model construction, using the PLS marker variable method to detect and control for common method variance (69). As a result, the addition of marker variables does not significantly alter either the Beta (*β*) value (differences between 0.000 and 0.071) or the *R*^2^ changes (the difference between 0.000 and 0.026). As a result, it can be said that this study’s CMV is not a significant problem.

This study collected a total of 375 available responses, and the respondent demographics are presented in Table 1 where the various categories of respondent demographics and their percentages can be viewed, along with the descriptive analysis of the instruments generated by SPSS version 27, including the gender, age, race, marital status, city, highest academic, occupation, income, working experience are the factors that will be examined.

**Table 1:** Respondents’ demographics.

Demographic data	Items	Frequency	Percent (%)
Gender	Male	167	44.5
	Female	208	55.5
Age	60–65 years old	89	23.7
	66–70 years old	129	34.4
	71–80 years old	120	32.0
	81 years old and above	37	9.9
Education qualification	Secondary school and below	245	65.3
	College	83	22.1
	Undergraduate	46	12.3
	Master’s degree	1	0.3
	PhD	0	0
Respondent’s income	Less than ¥1,000	4	1.1
	¥1,001 to ¥2000	9	2.4
	¥2001 to ¥3,000	74	19.7
	¥3,001 to ¥4,000	122	32.5
	¥4,001 to ¥5,000	73	19.5
	Above ¥5,001	93	24.8
Physical condition	Never felt uncomfortable	71	18.9
	Occasional discomfort	277	73.9
	Frequent discomfort	27	7.2
How many children will support you in your old age	0	10	2.7
	1	298	79.5
	2	56	14.9
	3	9	2.4
	4	1	0.3
	Above 4	1	0.3

Source: SPSS data analysis results.

*n* = 375 (after being deleted by Straight Lining).

#### 4.2.2 Assessment of the measurement model

This research’s measurement model was assessed in four steps: indicator reliability, internal consistency reliability, convergent validity, and discriminant validity. The assessment is followed by the guidelines of Hair et al. (54).

First, Indicator reliability explains the commonality of an item or indicator. If an indicator’s outer loading value is more than 0.7, it is considered reliable since the structure explains at least 50% of the variance in the indicator (54). After the PLS algorithm, it was found that the outer loading of the SR1 measurement term in the RS variable was −0.251and SR2 measurement term in the RS variable was −0.284, which was removed and then the PLS algorithm was performed again, as shown in Table 2, the outer loadings of all measurement items range from 0.844 to 0.971, indicating good indicator reliability.

**Table 2:** Measurement model for the constructs.

Constructs	Items	Indicator reliability	Convergent validity	Internal consistency reliability	
		Outer loadings	Average variance extracted (AVE)	Composite Reliability (CR)(rho_c)	Cronbach’s alpha
Attitude	ATT1	0.946	0.886	0.969	0.957
	ATT2	0.955			
	ATT3	0.921			
	ATT4	0.942			
Effort expectancy	EE1	0.814	0.765	0.929	0.898
	EE2	0.902			
	EE3	0.913			
	EE4	0.866			
Facilitating conditions	FC1	0.838	0.753	0.924	0.898
	FC2	0.888			
	FC3	0.861			
	FC4	0.882			
Financial risk	FR1	0.889	0.798	0.941	0.916
	FR2	0.905			
	FR3	0.919			
	FR4	0.86			
Hedonic motivation	HM1	0.852	0.832	0.952	0.932
	HM2	0.934			
	HM3	0.929			
	HM4	0.931			
Intergenerational emotional support	IES1	0.921	0.84	0.94	0.904
	IES2	0.935			
	IES3	0.892			
Intergenerational financial support	IFS1	0.87	0.809	0.927	0.884
	IFS2	0.93			
	IFS3	0.898			
Intention to use	IU1	0.886	0.846	0.956	0.939
	IU2	0.934			
	IU3	0.936			
	IU4	0.922			
Life satisfaction	LS1	0.93	0.877	0.956	0.93
	LS2	0.946			
	LS3	0.934			
Performance expectancy	PE1	0.843	0.812	0.945	0.922
	PE2	0.938			
	PE3	0.931			
	PE4	0.891			
Performance risk	PFR1	0.843	0.791	0.938	0.914
	PFR2	0.924			
	PFR3	0.883			
	PFR4	0.906			
Privacy risk	PR1	0.931	0.902	0.974	0.964
	PR2	0.945			
	PR3	0.971			
	PR4	0.952			
Perceived trust	PT1	0.912	0.873	0.965	0.951
	PT2	0.945			
	PT3	0.945			
	PT4	0.934			
Price value	PV1	0.888	0.761	0.927	0.894
	PV2	0.889			
	PV3	0.913			
	PV4	0.795			
Social influence	SI1	0.921	0.843	0.955	0.938
	SI2	0.931			
	SI3	0.9			
	SI4	0.919			
Social risk	SR3	0.956	0.924	0.961	0.919
	SR4	0.967			
Technology anxiety	TA1	0.788	0.762	0.927	0.898
	TA2	0.844			
	TA3	0.923			
	TA4	0.928			

Source: SmartPLS data analysis results.

Next, Cronbach’s *α* scores and composite reliability (CR) were used in the study to evaluate the constructs’ internal consistency validity. The CR suggests that the acceptable value for reliability is above 0.7 (55). This study’s Cronbach’s α scores range from 0.924 to 0.974, meeting the minimum threshold value of 0.7 (shown in Table 2).

The convergent validity was then examined using the average variance extracted (AVE). When a construct’s AVE value is greater than 0.50, it is considered to have sufficient convergent validity because it can account for at least 50% of the variation of its items (54). Table 2 demonstrates all AVEs above 0.50, with a range of 0.753–0.886, showing that the convergent validity of all constructs is within an acceptable range.

Furthermore, discriminant validity assessment is the final stage of measurement model evaluation. This research used the Fornell–Larcker’s criterion, Cross Loading, and Heterotrait–Monotrait (HTMT) criterion to determine discriminant validity. As the cross-loading table in Table 2 shows, in this study, all the constructs themselves are well loaded into their own constructs, and their loading values are greater than the loading values of the measured items with other constructs, so there is no cross-loading between the constructs in this study model, which also provides support for the discriminant validity of the model. According to the criteria established by Dijkstra and Henseler (56), the model has discriminant validity if the HTMT is smaller than 0.9. The HTMT values for all constructs were below 0.9, indicating that the model had discriminant validity.

#### 4.2.3 Assessment of the structural model

After the measurement model has been assessed, the structural model should be evaluated using the following procedures: collinearity assessment, path coefficient, coefficient of determination (*R*^2^), effect size (*f*^2^), predictive relevance (*Q*^2^), model fit indices, PLS prediction, and hypothesis testing.

The VIF value should not exceed 5 (54). As shown in Table 3, the VIF of all constructs in the internal model of this study is less than 5, and their VIF values range from 1.072 to 4.374. The results indicate that the degree of covariance in this model is within the acceptable range.

**Table 3:** Values of the variation inflation factor for collinearity.

Variables	ATT	IU	LS	PT
ATT		3.78		
EE	2.66			
FC	4.374			
FR				1.957
HM	3.938			
IES			1.37	
IFS			1.37	
IU				
LS		4.02		
PE	2.234			
PFR				2.685
PR				2.526
PT		4.263		
PV	3.194			
SI	3.294			
SR				1.072
TA		1.296		

As a rule, *R*^2^ values below 0.25 indicate very weak, whereas 0.75, 0.50, and 0.25 indicate substantial, moderate, and weak values, respectively (55). ATT, IU, LS, and PT were the endogenous variables, and their respective *R*^2^ values were 0.562 (moderate), 0.725 (moderate), 0.437 (weak), and 0.423 (weak). Suggested standards for estimating effect size are *f*^2^ ≥ 0.02, *f*^2^ ≥ 0.15, and *f*^2^ ≥ 0.35, which equate to small, medium, and large effect sizes of exogenous constructs, respectively (57). The effect size *f*^2^ was from 0.001 to 0.632 (see Table 4).

**Table 4:** Coefficient of determination *R*^2^.

Variables	*R* ^2^	Consideration
ATT	0.562	Moderate
IU	0.725	Moderate
LS	0.437	Weak
PT	0.423	Weak

Predictive Relevance (*Q*^2^) is evaluated as the structural model’s fourth assessment (70). The fact that the *Q*^2^ values for this research’s structural model were greater than zero means that the model’s explanatory and predictive power was significant (58). The endogenous constructs of ATT were 0.492, IU were 0.601, LS were 0.379, and PT were 0.362 (see Table 5).

**Table 5:** Results of predictive relevance (*Q*^2^).

Variables	*Q* ^2^	Indicates predictive relevance
ATT	0.492	Medium
IU	0.601	Large
LS	0.379	Medium
PT	0.362	Medium

Model fit was evaluated using several global fit indices. The saturated model demonstrated excellent fit, with an SRMR of 0.041, well below the recommended cutoff of 0.08 (59). Both discrepancy measures—d_ULS (3.381) and d_G (2.296)—fell well within the bootstrapped confidence intervals, indicating that the model adequately reproduces the empirical covariance structure (60). Although the Chi-square statistic was relatively large (*χ*^2^ = 4874.358), this index is known to be highly sensitive to sample size and is therefore not considered a reliable standalone indicator of fit in PLS-SEM and SEM contexts. The NFI value of 0.824 exceeds the commonly referenced threshold of 0.80 for acceptable fit in complex structural models and is consistent with previous recommendations for PLS-SEM applications (55). Taken together, these results indicate that the model achieves satisfactory global fit and is appropriate for further interpretation of the structural relationships.

Lastly, the PLS predicted assessed the predictive power of IU. Based on Table 6, each indicator’s RMSE values are lower than the naive linear regression model (LM) benchmark. According to (61), the model has high predictive power.

**Table 6:** Hypothesis testing.

Hypothesis	Relationship	Std. beta	Standard deviation (Std. error)	*T*-statistics	*p*-values	Supported
H1	PE → ATT	0.152	0.058	2.608	0.005	Yes
H2	EE → ATT	−0.076	0.069	1.105	0.135	No
H3	SI → ATT	0.225	0.077	2.923	0.002	Yes
H4	FC → ATT	0.076	0.086	0.884	0.188	No
H5	HM → ATT	0.295	0.092	3.214	0.001	Yes
H6	PV → ATT	0.158	0.08	1.975	0.024	Yes
H7	FR → PT	−0.089	0.073	1.217	0.112	No
H8	PFR → PT	0.04	0.077	0.518	0.302	No
H9	PR → PT	−0.205	0.077	2.644	0.004	Yes
H10	SR → PT	0.625	0.042	15.047	<0.001	No
H11	IFS → LS	0.026	0.045	0.582	0.28	No
H12	IES → LS	0.647	0.049	13.175	<0.001	Yes
H13	ATT → IU	0.316	0.059	5.322	<0.001	Yes
H14	PT → IU	0.303	0.066	4.589	<0.001	Yes
H15	LS → IU	0.275	0.063	4.396	<0.001	Yes

Source: SmartPLS data analysis results.

To examine the significance level of the paths, the bootstrapping function of Smart PLS 4.0 was employed to obtain t-statistics for each path. The bootstrap procedure was configured with a significance level of 0.05, a one-tailed test, and 10,000 bootstrap subsamples (54). The detailed results of the hypothesis testing are presented in Table 6.

**Figure 3:**
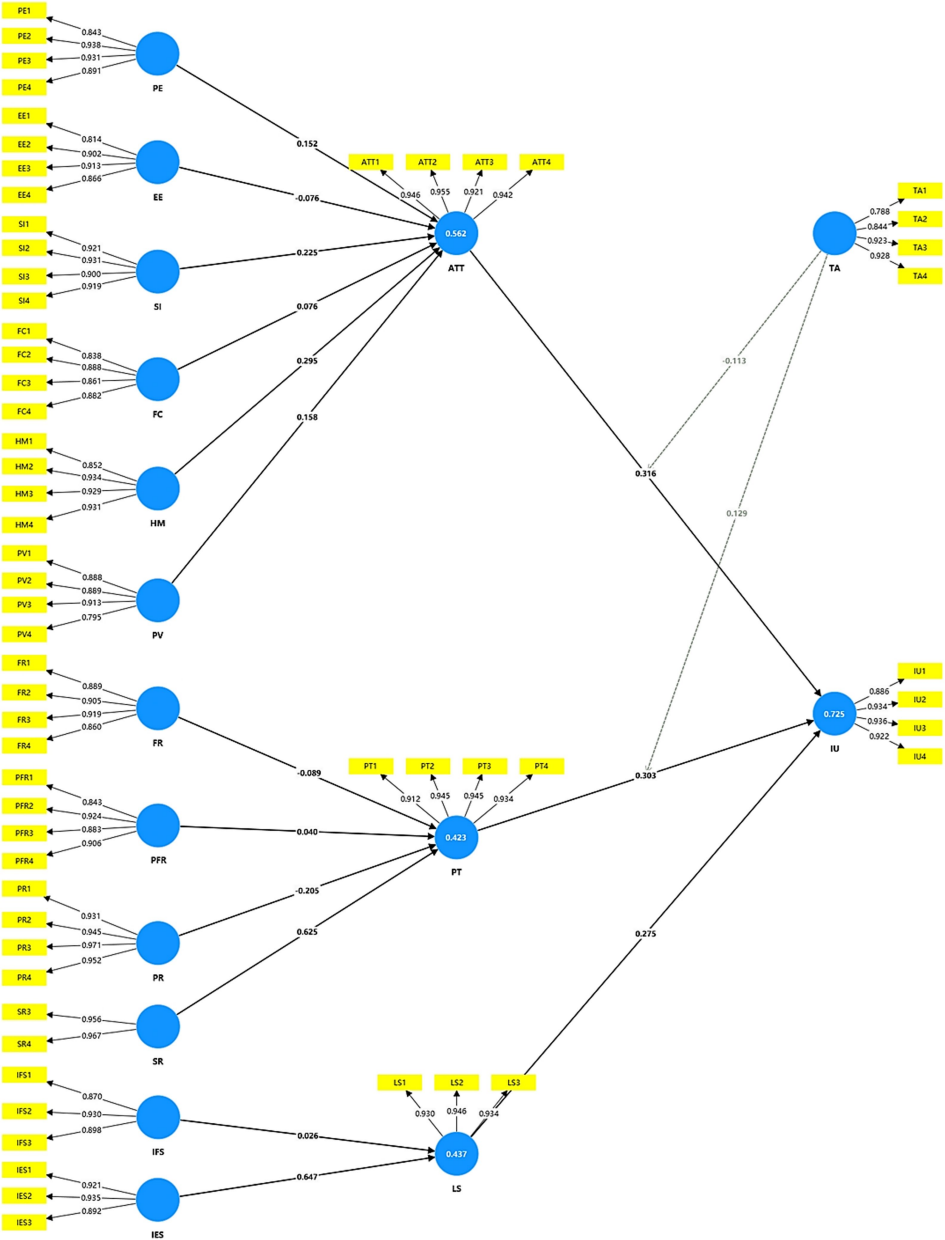
Hypothesis testing results.

H1, H2, H3, H4, H5, and H6 are the hypotheses about the potential critical factors that affect attitude. The research shows that H1 is supported because of the results (*β* = 0.152, *t* = 2.608, *p* (0.005) < 0.01). Meanwhile, H3 (β = 0.225, *t* = 2.923, *p* (0.002) < 0.01), H5 (β = 0.295, *t* = 3.214, *p* (0.001) < 0.01) and H6 (β = 0.158, *t* = 1.975, *p* (0.024) < 0.01) both are supported. The research shows that H2 is not supported because the result “*p*” is bigger than 0.05 (β = −0.076, *t* = 1.105, *p* (0.135) > 0.05). H4 is also not supported because the result “p” is bigger than 0.05 (β = 0.076, *t* = 0.884, *p* (0.188) > 0.05). H7, H8, H9, and H10 test whether the perceived risks affect perceived trust. The research shows that H7 is not supported because the result “*p*” is bigger than 0.05 (β = −0.089, *t* = 1.217, *p* (0.112) > 0.05). H8 (β = 0.04, *t* = 0.518, *p* (0.302) > 0.05) and H10 (β = 0.625, *t* = 15.047, *p* < 0.001) are not supported because the result shows positive relationships between perceived risks and perceived trust. H9 is supported because of the results (β = −0.205, *t* = 2.644, *p* (0.004) < 0.01). H11 and H12 test the relationship between the intergenerational support factors and life satisfaction. The research shows that H11 is not supported because the result “p” is bigger than 0.05 (β = 0.026, *t* = 0.582, *p* (0.28) > 0.05). H12 is supported because of the results (β = 0.647, *t* = 13.175, p < 0.001). H13, H14, and H15 are the hypotheses about the factors influencing intention to use. The research shows that H13 is supported because of the results (β = 0.316, *t* = 5.322, p < 0.001). Meanwhile, H14 (β = 0.303, *t* = 4.589, p < 0.001) and H15 (β = 0.275, *t* = 4.396, *p* (0.000) < 0.01) both are supported (see Figure 3).

## 5 Discussion

This study reveals a multi-dimensional mechanism driving usage intention, with attitude emerging as the most potent predictor—alongside perceived trust and life satisfaction, all of which positively boost usage intention. Attitudes themselves are shaped by four key factors: performance expectancy, social influence, hedonic motivation, and price value, while effort expectancy and facilitating conditions exert no significant impact. In terms of perceived trust, only privacy risk undermines it, whereas financial, performance, and social risks prove irrelevant. For life satisfaction, emotional support serves as a constructive factor, while financial support offers no meaningful contribution. Additionally, technology anxiety intensifies the negative influence of attitude on usage intention but does not alter the role of perceived trust in this relationship.

### 5.1 Performance expectancy, social influence, hedonic motivation, and price value drive positive attitudes; effort expectancy and facilitating conditions have no impact

This study found that performance expectancy (H1), social influence (H3), hedonic motivation (H5), and price value (H6) show positive correlations with the attitudes of older people toward smart elderly care applications. Specifically, performance expectancy emerged as a key determinant, as older people prioritize the functional benefits and improvements in their quality of life associated with such technologies (22, 23). Social influence also plays a pivotal role, with support from family members and friends contributing significantly to favorable attitudes (4, 13). Moreover, hedonic motivation positively influences attitudes, underscoring the importance of enjoyment and emotional satisfaction in technology acceptance among older individuals (62). Price value is another significant factor, as older people are more likely to develop favorable attitudes toward applications that are perceived as offering good value for money (50, 63).

Conversely, effort expectancy (H2) and facilitating conditions (H4) have no significant positive association with attitudes. A possible explanation for this research finding is the mature development of various applications in China, such as social applications like WeChat, a popular mobile platform in China, that offers messaging, toll-free calls, information browsing, and mobile payments, with 79.87% of older adults using it as their primary social tool (4). Older people in Chinese cities have already become accustomed to using and operating these applications, which is why effort expectancy and facilitating conditions are not significant factor influencing their attitude toward the usage of smart elderly care applications.

### 5.2 Only privacy risk reduces perceived trust; financial, performance, and social risks are insignificant

The findings reveal that among the four types of perceived risks, only privacy risk (H9) shows a negative correlation with perceived trust. Privacy concerns are particularly salient for older people, given the sensitive nature of personal data involved in smart elderly care applications, such as health status, home addresses, and family information (66). The potential misuse of such data heightens privacy-related apprehensions, making privacy risk the most influential factor affecting trust in this context (28, 41, 64).

In contrast, financial risk (H7), performance risk (H8), and social risk (H10) have no significant positive association with perceived trust. These findings align with previous studies suggesting that the mature digital payment environment in China, coupled with stringent regulatory protections, has effectively minimized financial concerns among older consumers (40). Similarly, performance risk appears to be mitigated by the availability of online reviews and third-party evaluations, which assist users in making informed decisions. Social risk is also not significant, possibly due to the personalized and private nature of elderly care services, which reduces concerns about reputational consequences.

### 5.3 Emotional support enhances life satisfaction; no significant positive association between financial support and life satisfaction

The study confirms that intergenerational emotional support (H12) has a significant positive correlation with life satisfaction among older people, whereas intergenerational financial support (H11) have no significant positive association. Emotional support, encompassing companionship, care, and emotional closeness from children, emerges as a crucial determinant of life satisfaction (20, 42). This finding suggests that emotional connections outweigh financial assistance in enhancing older people’s well-being and overall quality of life.

The lack of a significant relationship between intergenerational financial support and life satisfaction may be attributed to the specific context of this study. The sample date for this study was collected from Shanghai—the most rapidly aging first-tier city in China—and Nantong, the most severely aging second-tier city. Both cities are situated in economically advanced regions, with Shanghai serving as the nation’s economic hub. Due to the high level of economic development in these areas, elderly users often receive higher pensions and enjoy more substantial social welfare benefits. Consequently, their reliance on financial support from younger generations is relatively low, which may explain why Financial Support does not emerge as a significant determinant of life satisfaction in this context.

### 5.4 Attitude, perceived trust, and life satisfaction all boost usage intention

This study demonstrates that attitude (H13), perceived trust (H14), and life satisfaction (H15) all positively correlated with the intention to use smart elderly care applications. Among these factors, attitude is identified as the most influential predictor of usage intention, underscoring its central role in technology adoption among older people (23, 62). A more favorable attitude toward the technology directly translates into a stronger intention to use it.

Having discussed the factors influencing attitudes, we now turn to perceived trust and risk perceptions, which further shape older people. Perceived trust also plays a significant role in shaping usage intentions. Trust in both the technological functionality and security of the applications is essential for older people, particularly given their concerns regarding privacy and usability (28, 41, 46). Additionally, life satisfaction positively affects usage intention. Older people with higher life satisfaction, often resulting from strong intergenerational support, exhibit greater willingness to adopt smart elderly care applications, as they feel more confident and supported in navigating digital technologies (20, 42).

### 5.5 Technology anxiety strengthens negative impact of attitude on usage intention, but does not affect perceived trust’s role

The findings of this study reveal that the technology anxiety weakens the positive relationship between ATT and IU, thus supporting Hypothesis 16. This suggests that technology anxiety serves as a critical moderating factor in the adoption of smart technologies among older people. Hung (50) observed that when new technologies are perceived as overly complex or difficult to use, older individuals are more likely to reject them to avoid feelings of anxiety. Consistent with these findings, Jeng et al. (27) empirically validated the moderating role of technology anxiety in the relationship between attitude and behavioral intention, further validating its role in technology acceptance models involving older populations.

However, the results of this study do not support the hypothesis that technology anxiety strengthens the negative relationship between perceived trust and the intention to use smart elderly care applications. Although Dekkal et al. (45) reported that technology anxiety may negatively moderate the relationship between trust and usage intention, they also cautioned that this effect was only marginally significant and should be interpreted with care. The result contradicts our prediction, which may be attributed to a broader sense of helplessness or lack of control over technological interactions that discourages engagement with the technology altogether. Moreover, in real-world scenarios, technology anxiety often manifests at the point of use. If perceived trust in the application is not established early on, older adults may choose not to engage with the system at all—thereby precluding the possibility for technology anxiety to exert any moderating influence. As such, the absence of perceived trust may already be sufficient to deter usage, independent of anxiety levels, explaining the non-significant moderating effect observed in this study.

## 6 Theoretical contributions

Theoretically, this study extension of the UTAUT2 model incorporating perceived risks, intergenerational support, and technology anxiety. First, by integrating perceived risk dimensions into the UTAUT2 model, it extends the theoretical framework and provides deeper insights into how these factors shape technology adoption in this demographic. Second, it incorporates intergenerational support to address a key limitation in prior research on elderly care, which often examined older individuals in isolation, thereby highlighting the broader influence of family dynamics and intergenerational relationships. Third, it introduces technology anxiety as a moderating variable and demonstrates its critical role in shaping technology acceptance, offering valuable theoretical implications for smart elderly care adoption. Fourth, it expands the application of the UTAUT2 model to the context of smart elderly care in China. The model’s explanatory power is confirmed by a *R*^2^ value of 72.5% for the dependent variable “Intention to Use” underscoring its robustness in capturing adoption intentions for smart elderly care applications. Moreover, given that the COVID-19 pandemic heightened the urgency of addressing older people’s care needs, the study provides a timely Chinese sample that advances smart elderly care research in the post-pandemic era. It also offers valuable insights for Asian countries with similar cultural backgrounds and developing nations at comparable economic levels, shedding light on the localized adaptation and promotion of smart elderly care practices across diverse contexts.

This study identifies key determinants of older people’s intention to use smart elderly care applications, providing actionable insights for stakeholders to boost adoption. For application designers/developers, prioritize practicality (e.g., daily care, health monitoring, communication), integrate social features (e.g., expert advice, family interactions) and entertainment modules (e.g., games, music), adopt user-friendly designs (e.g., step-by-step guides) to mitigate technological anxiety, and ensure compatibility with smartphones and high-traffic applications. For providers/companies, enhance promotion via experts, social media, and community outreach, safeguard privacy through biometric recognition and data protection, and offer cost-effective service packages to build trust and cater to price sensitivity. For governments, encourage industry development via national demonstrations and unified service platforms, refine elderly capability assessment standards, and strengthen privacy protection through regulations to foster a secure ecosystem. For users, intergenerational emotional support to improve life satisfaction and elderly users’ proactive adoption of new technologies will further drive acceptance, especially as digitally literate “internet generation” individuals enter old age.

## 7 Conclusion, limitations, and future studies

This study makes an original contribution by integrating perceived risk, intergenerational support, attitudes, perceived trust, and life satisfaction within a unified theoretical framework. It also explicitly incorporates the moderating role of technology anxiety, thereby accounting for both the psychological characteristics of older people and the unique family dynamics in the Chinese context. The proposed model offers new insights into the behavioral mechanisms underlying older people’s adoption of smart elderly care technologies, with important implications for both theory and practice in aging societies. These findings have important theoretical implications. First, by integrating perceived risk dimensions, intergenerational support, and technology anxiety—elements particularly relevant to older Chinese users—into the UTAUT2 model, this study deepens our understanding of how such factors affect technology adoption within this demographic. Second, the findings underscore the importance of considering psychological traits and cultural context when applying technology acceptance models across different populations and environments. Third, this study expands the research on the UTAUT2 model within the context of smart elderly care applications in China, offering a localized perspective that contributes a “China solution” for developing countries, particularly those in Asia. For stakeholders, this study suggests several strategies to enhance the adoption of smart elderly care applications.

Despite its contributions, this study has limitations that provide directions for future research. First, as a cross-sectional study focusing on usage intention rather than actual behavior, it captures only a snapshot of the adoption process and cannot infer causality. Second, the sample is restricted to first- and second-tier Chinese cities, limiting generalizability to rural or less developed regions. Third, the questionnaire was administered online rather than through face-to-face interaction, which may have excluded elderly users with limited access to or experience with digital technologies. Additionally, self-reported data is prone to social desirability bias (see Table 7).

**Table 7:** Results of the moderator analysis.

Hypothesis	Relationship	Std. beta	Sample mean (M)	Standard deviation (STDEV)	*T* statistics (|O/STDEV|)	*P*-values	*f* ^2^	Result
H16	TA * ATT → IU	−0.113	−0.107	0.056	2.036	0.021	0.1004	Supported
H17	TA * PT → IU	0.129	0.121	0.053	2.446	0.007	0.0599	Not Supported

Source: SmartPLS data analysis results.

TA*ATT (*β* = −0.113, *t*-value = 2.036, *p* = 0.021 < 0.1) significantly moderated the relationship between attitude and intention to use. Therefore, hypothesis H16 was supported. H17 was not supported because the observed interaction is positive (*β* = 0.129, *t*-value = 2.446, *p* = 0.007 < 0.1), contrary to the hypothesized negative direction.

To address these limitations, future studies should prioritize three key directions aligned with targeted research needs: First, employ longitudinal designs to systematically track actual usage behavior and sustained adoption over an extended period, enabling the inference of causal relationships. Second, explore regional differences by expanding the sample frame to include third- and fourth-tier cities, rural areas, and less developed regions, thereby enhancing the generalizability of research findings. Third, incorporate objective measures of technology use (e.g., device usage logs, platform interaction data) to complement or replace self-reported data, mitigating the impact of social desirability bias. Furthermore, future research could integrate investigations into the moderating roles of demographic characteristics, family structure, and health-related variables to deepen the understanding of technology adoption mechanisms across diverse populations (see Table 8).

**Table 8:** The list of questionnaire items.

Strongly disagree	Disagree	Neutral	Agree	Strongly agree				
1	2	3	4	5				
	Performance Expectancy (PE)							
1	I find the smart elderly care applications useful in my daily life.			1	2	3	4	5
2	Using smart elderly care applications increases my chances of achieving things that are important to me.			1	2	3	4	5
3	Using smart elderly care applications helps me accomplish things more quickly.			1	2	3	4	5
4	Using the smart elderly care applications has increased my efficiency.			1	2	3	4	5

	Effort Expectancy (EE)					
5	Learning how to use smart elderly care applications is easy for me.	1	2	3	4	5
6	My interaction with the smart elderly care applications is clear and understandable (e.g., Button operation, service search, online payment, device connection, etc.).	1	2	3	4	5
7	I find smart elderly care applications easy to use.	1	2	3	4	5
8	It is easy for me to become skillful at using smart elderly care applications.	1	2	3	4	5
	Social Influence (SI)					
9	People who are important to me would think that I should use smart elderly care applications.	1	2	3	4	5
10	People who are familiar with me think that I should use smart elderly care applications.	1	2	3	4	5
11	People who influence my behavior think that I should use smart elderly care applications.	1	2	3	4	5
12	People whose opinions I value would like me to use the smart elderly care applications.	1	2	3	4	5
	Facilitating Conditions (FC)					
13	I have the resources necessary to use smart elderly care applications (e.g., smartphones, Internet broadband, etc.).	1	2	3	4	5
14	I have the knowledge necessary to use smart elderly care applications.	1	2	3	4	5
15	Smart elderly care applications are compatible with other technologies I use.	1	2	3	4	5
16	I can get help from others when I have difficulties using smart elderly care applications.	1	2	3	4	5

	Hedonic Motivation (HM)					
17	I think it would be fun if I used smart elderly care applications.	1	2	3	4	5
18	It would be enjoyable if I used smart elderly care applications	1	2	3	4	5
19	I think it would be very entertaining if I used smart elderly care applications.	1	2	3	4	5
20	Using the smart elderly care applications would be comfortable.	1	2	3	4	5

	Price Value (PV)					
21	Smart elderly care applications are a good value for the money.	1	2	3	4	5
22	Smart elderly care applications offer the amount corresponding to the money you pay.	1	2	3	4	5
23	I find it economical to use smart elderly care applications.	1	2	3	4	5
24	Irrespective of price, smart elderly care applications are always a good deal.	1	2	3	4	5

	Financial risk (FR)					
25	Using smart elderly care applications could be subject to potential fraud.	1	2	3	4	5
26	Using smart elderly care applications could be subject to financial risk.	1	2	3	4	5
27	What are the chances that you stand to lose money if you use smart elderly care applications?	1	2	3	4	5
28	Signing up for and using smart elderly care applications would lead to a financial loss for me (e.g., electronic wallet theft, phishing websites, payment password leakage, etc.).	1	2	3	4	5

	Performance risk (PFR)					
29	The smart elderly care applications do not work as expected.	1	2	3	4	5
30	The performance level of smart elderly care applications may be lower than designed.	1	2	3	4	5
31	The service performance of smart elderly care applications may not match their advertised level.	1	2	3	4	5
32	The smart elderly care app system may be unstable or blocked.	1	2	3	4	5

	Privacy risk (PR)					
33	Privacy information could be misused, inappropriately shared, or sold when using smart elderly care applications.	1	2	3	4	5
34	Payment information could be collected, tracked, and analyzed using smart elderly care applications.	1	2	3	4	5
35	Privacy could be exposed or accessed when using smart elderly care applications.	1	2	3	4	5
36	Personal information could be intercepted or accessed when using smart elderly care applications.	1	2	3	4	5

	Social risk (SR)					
37	When my smart elderly care applications incur fraud, I will have a potential loss of status in one’s social group (e.g., authority, discourse power, dominance, etc.).	1	2	3	4	5
38	I’m sure that if I decided to use smart elderly care applications and have operational errors, my friends and family would think less of me.	1	2	3	4	5
39	I believe that people (friends and family) who influence my behavior think that I must use smart elderly care applications.	1	2	3	4	5
40	I believe that people (friends and family) who are important to me strongly support the use of smart elderly care applications.	1	2	3	4	5

	Intergenerational financial support (IFS)					
41	My children have supported my living expenses.	1	2	3	4	5
42	My children have supported my financial cost of using the smart elderly care applications.	1	2	3	4	5
43	My children gave me food or gifts to ease the financial stress of using smart elderly care applications.	1	2	3	4	5

	Intergenerational emotional support (IES)					
44	I get along well with my children when I use smart elderly care applications.	1	2	3	4	5
45	My children are willing to listen when I talk about my concerns and problems with smart elderly care applications.	1	2	3	4	5
46	My children and I are close and can support me in using smart elderly care applications.	1	2	3	4	5

Strongly disagree	Disagree	Slightly disagree	Neutral	Slightly agree	Agree			Strongly agree			
1	2	3	4	5	6			7			
	Attitude (ATT)										
47	Using smart elderly care applications is a good idea.				1	2	3	4	5	6	7
48	I feel that using smart elderly care applications is pleasant.				1	2	3	4	5	6	7
49	In my opinion, it is desirable to use smart elderly care applications.				1	2	3	4	5	6	7
50	In my view, using smart elderly care applications is a wise idea.				1	2	3	4	5	6	7

	Perceived Trust (PT)							
51	I trust that smart elderly care applications are safe and have reliable features.	1	2	3	4	5	6	7
52	I trust transactions done by smart elderly care applications.	1	2	3	4	5	6	7
53	I trust smart elderly care applications to keep my financial information secure.	1	2	3	4	5	6	7
54	I trust smart elderly care applications to keep my personal information safe.	1	2	3	4	5	6	7

	Life Satisfaction (LS)							
55	My life is close to the idea, which motivates me to try smart elderly care applications.	1	2	3	4	5	6	7
56	My living conditions are very good, which motivates me to try smart elderly care applications.	1	2	3	4	5	6	7
57	I am very satisfied with my life, which motivates me to try smart elderly care applications.	1	2	3	4	5	6	7

	Technology anxiety (TA)							
58	I feel apprehensive about using smart elderly care applications.	1	2	3	4	5	6	7
59	I have avoided the smart elderly care applications because they are unfamiliar to me.	1	2	3	4	5	6	7
60	I hesitate to use smart elderly care applications for fear of making mistakes I cannot correct.	1	2	3	4	5	6	7
61	Technical terms of smart elderly care applications sound like confusing jargon to me.	1	2	3	4	5	6	7

	Intention to use (IU)							
62	I intend to use smart elderly care applications in the future.	1	2	3	4	5	6	7
63	I will always try to use smart elderly care applications in my daily life.	1	2	3	4	5	6	7
64	I plan to use smart elderly care applications frequently.	1	2	3	4	5	6	7
65	I will recommend others to use smart elderly care applications.	1	2	3	4	5	6	7

	General Questions (Maker Variable)							
66	I often change my mind.	1	2	3	4	5	6	7
67	Once I have concluded, I am not likely to change my mind.	1	2	3	4	5	6	7
68	I do not change my mind easily.	1	2	3	4	5	6	7
69	My views are very consistent over time.	1	2	3	4	5	6	7

## Data Availability

The original contributions presented in the study are included in the article/supplementary material, further inquiries can be directed to the corresponding authors.
